# Silent hypoxia: higher NO in red blood cells of COVID-19 patients

**DOI:** 10.1186/s12890-020-01310-8

**Published:** 2020-10-16

**Authors:** Esmaeil Mortaz, Majid Malkmohammad, Hamidreza Jamaati, Parisa Adimi Naghan, Seyed MohamadReza Hashemian, Payam Tabarsi, Maohammad Varahram, Hamidreza Zaheri, Efsun Gonca Uğur Chousein, Gert Folkerts, Ian M. Adcock

**Affiliations:** 1 Mycobacteriology Research Center, National Research Institute of Tuberculosis and Lung Diseases (NRITLD), Masih Daneshvari Hospital, Shahid Beheshti University of Medical Sciences, Tehran, Iran; 2 Tracheal Disease Research Center, National Research Institute of Tuberculosisand Lung Diseases (NRITLD), Shahid Beheshti University of Medical Science, Tehran, Iran; 3 Chronic Respiratory Diseases Research Center, National Research Institute ofTuberculosis and Lung Diseases (NRITLD), Shahid Beheshti University of Medical Sciences, Tehran, Iran; 4 University of Health Sciences Turkey, Yedikule Chest Diseases and Thoracic Surgery, Education and research Hospital, Department of pulmonology, Istanbul, Turkey; 5 Division of Pharmacology, Utrecht Institute for Pharmaceutical Sciences, Faculty of Science, Utrecht University, Utrecht, Netherlands; 6 Cell and Molecular Biology Group, Airways Disease Section, Faculty of Medicine, National Heart and Lung Institute, Imperial College London, London, UK; 7 Priority Research Centre for Asthma and Respiratory Disease, Hunter Medical Research Institute, University of Newcastle, Newcastle, NSW Australia

**Keywords:** COVID-19, NO, Hypoxia

## Abstract

**Background:**

Severe acute respiratory syndrome coronavirus 2 (SARS-CoV-2) that causes coronavirus disease 2019 (COVID-19) has spread to almost 100 countries, infected over 31 M patients and resulted in 961 K deaths worldwide as of 21st September 2020. The major clinical feature of severe COVID-19 requiring ventilation is acute respiratory distress syndrome (ARDS) with multi-functional failure as a result of a cytokine storm with increased serum levels of cytokines. The pathogenesis of the respiratory failure in COVID-19 is yet unknown, but diffuse alveolar damage with interstitial thickening leading to compromised gas exchange is a plausible mechanism. Hypoxia is seen in the COVID-19 patients, however, patients present with a distinct phenotype. Intracellular levels of nitric oxide (NO) play an important role in the vasodilation of small vessels. To elucidate the intracellular levels of NO inside of RBCs in COVID-19 patients compared with that of healthy control subjects.

**Methods:**

We recruited 14 COVID-19 infected cases who had pulmonary involvement of their disease, 4 non-COVID-19 healthy controls (without pulmonary involvement and were not hypoxic) and 2 hypoxic non-COVID-19 patients subjects who presented at the Masih Daneshvari Hospital of Tehran, Iran between March–May 2020. Whole blood samples were harvested from patients and intracellular NO levels in 1 × 10^6^ red blood cells (RBC) was measured by DAF staining using flow cytometry (FACS Calibour, BD, CA, USA).

**Results:**

The Mean florescent of intensity for NO was significantly enhanced in COVID-19 patients compared with healthy control subjects (*P* ≤ 0.05). As a further control for whether hypoxia induced this higher intracellular NO, we evaluated the levels of NO inside RBC of hypoxic patients. No significant differences in NO levels were seen between the hypoxic and non-hypoxic control group.

**Conclusions:**

This pilot study demonstrates increased levels of intracellular NO in RBCs from COVID-19 patients. Future multi-centre studies should examine whether this is seen in a larger number of COVID-19 patients and whether NO therapy may be of use in these severe COVID-19 patients.

## Background

The coronavirus (severe acute respiratory syndrome coronavirus 2, SARS-CoV-2) that causes coronavirus disease 2019 (COVID-19) has spread to almost 100 countries, infected over 31 million patients and resulted in 961 K deaths worldwide as of 21st September 2020 [1]. The major clinical feature is acute respiratory distress syndrome (ARDS) with a key complication being heart and multi-functional failure. Normal blood oxygen saturation levels are at least 95% and this decreases in most lung diseases including pneumonia and is further decreased in the presence of stiff or oedematous lungs whilst increasing levels of carbon dioxide are usually seen in COVID-19 patients with pneumonia [2]. Thus, many COVID-19-infected patients with pulmonary involvement have hypoxia and dyspnea as important hallmarks of disease. Many COVID-19 patients are often alert and feeling relatively well and can easily talk despite the respiratory system being unable to sufficiently oxygenate blood a state known as ‘happy’ or silent hypoxia’ [3].

Red blood cells (RBCs) are highly adapted cells for blood gas transport. At the high oxygen tensions (*P*O2) prevailing in the pulmonary system, the blood is normally completely saturated with oxygen and hemoglobin (Hb) will formed an R structure. When the blood enters the microcirculation, the *P*O2 is attenuated promoting oxygen dissociation from hemoglobin and a shift to the T form [4].

Clinical examination of severe cases of COVID-19 patients shows a decreased ratio of arterial oxygen partial pressure to fractional inspired oxygen (PaO_2_:FiO_2_ ratio) with concomitant hypoxia and tachypnea in most cases [5]. Nitric oxide (NO) plays a key role in controlling the vascular system by regulating vascular tone and blood flow following activation of soluble guanylate cyclase (sGC) within the vascular smooth muscle. NO also controls mitochondrial oxygen consumption by inhibiting cytochrome *c* oxidase [6]. RBCs have long been considered as powerful scavengers of endothelial cell-derived NO, participating in systemic NO metabolism mainly by limiting NO bioavailability [7]. RBCs passing through the microcirculation sense tissue oxygen conditions via their degree of deoxygenation and couple this information to the release of vasodilatory compounds including ATP and NO to enhance blood flow to hypoxic tissues [8]. NO is a free radical and has a critical pathophysiological role in infectious diseases.

Intracellular NO within RBCs is derived from three sources: a) entry from the cell exterior by binding to the highly conserved *β*-globin chain cysteine 93 residue to form bioactive S-nitrosohemoglobin (SNO–Hb) [9], b) formation from nitrite entering RBC due to the reductive potential of deoxyhemoglobin [10] and c) intracellular production of NO by RBC derived from an active and functional eNOS-like enzyme (RBC NOS). RBC NOS is localized in the RBC membrane and cytoplasm and has similar properties to eNOS in terms of phosphorylation sites controlling enzymatic activity and its dependence on intracellular calcium and L-arginine concentrations for its activity [11].

Transfer of NO from SNO–Hb to the membrane-bound anion exchanger (AE1) is required for transfer of NO out of the RBC and is dependent on both the SNO–Hb state (T or R) and the SNO–Hb concentration. Therefore, the ability of SNO–Hb to transfer NO to AE1 or other proteins such as glutathione are limiting factors in respiratory efficiency. The kinetics and allosteric regulation of Hb nitrosylation by oxygen and pH are consistent with the physiologic mechanisms that modulate tissue blood flow, namely acidosis and hypoxemia and tissue hypoxia leads to NO generation by the RBC via SNO–protein transfer of NO activity [12]. In addition, insults such as cellular stress activates RBC NOS, leading to NO release and vasodilation of vessel segments under hypoxic conditions. Together, this supports a prominent role of RBC-derived NO in the regulation of local blood flow [13].

Therefore, the erythrocrine function of RBCs i.e. the release of bioactive molecules including NO, NO metabolites, and ATP are likely to be important in tissue protection and regulation of cardiovascular homeostasis by RBCs. Despite this clear role of NO in vasodilation, there is little evidence regarding the role of NO in COVID-19 particularly in silent hypoxic patients. To examine the hypothesis that NO is important in regulating vasodilation during hypoxia in these subjects we studied intracellular levels of NO in COVID-19 patients.

## Methods

We examined 14 COVID-19 infected cases who had lung involvement of their disease, 4 non-COVID-19 healthy controls (without lung involvement and who were not hypoxic) and 2 hypoxic non-COVID-19 patients subjects who presented at the Masih Daneshvari Hospital of Tehran, Iran between March–May 2020. All COVID-19 infected cases were diagnosed based on the World Health Organization (WHO) interim guidance. Patients were confirmed positive for COVID-19 nucleic acid in the respiratory samples via real-time reverse-transcriptase polymerase-chain-reaction (RT-PCR) or serum specific antibodies and chest imaging including chest X ray and CT sacn. Demographic data of all participants is presented in Table 1. Red blood cells were isolated from 3 ml whole blood cells with EDTA used as an anticoagulant. Whole blood was diluted 400-fold with FACS buffer (BSA and PBS) and then stained with the NO-specific probe, 4-amino-5-methylamino-2′, 7′-difluorofluorescein (DAF-FM DA) (BD Pharmingin, catalog 566,663, USA). Intracellular NO was detected by flow cytometry (FCM, BD FACS Calibour) using DAF-FM DA dye as a flurochrome. Following incubation of RBC cell suspensions for 30 min with the dye (10 μM) a fluorescent signal was detected that corresponded to the level of intracellular NO.

**Table 1:** Demographic information of all participants (COVID-19 and control groups)

IgG	IgM	RT-PCR COVID-19	Mortalility	Radialogy	Ventilation	PCO2	O2/S	Sex	AGE	
–	–	–	–	–	–	41	92/97	M	39	Control
–	–	–	–	–	–	40	98/97	M	65	Control
–	–	–	–	–	–	40	97/98	F	44	Control
–	–	–	–	–	–	42	94/98	F	54	Control
		+	+	++	MV	56	69/86	F	80	Patient 1
		+	+	+++	MV	28	34/69	M	77	Patient 2
		+	–	++	–	47	61/87	M	34	Patient 3
		+	+	++	MV	44	26/51	F	62	Patient 4
		+	+	++	MV	47	43/65	M	72	Patient 5
+	+	–	+	++	–	63	36/63	M	69	Patient 6
+	+	–	+	++	MV	47.5	47/82	M	60	Patient 7
+	+	–	–	++	–	55	48/80	M	41	Patient 8
+	+	–	–	++	MV	58.6	36/69	M	66	Patient 9
+	+	–	–	++	–	45	98/98	M	61	Patient 10
+	+	–	+	++	NIV	41.4	81/82.5	F	74	Patient 11
+	+	–	–	++	–	41	40.5/74.6	F	58	Patient 12
+	+	–	–	++	–	44	84/97	F	81	Patient 13
–	–	+	+	++	MV	49	44/78	F	21	Patient 14

Abbreviation used: *MV* Mechanical Ventilation, *NIV* Non-invasive Ventilation

In radiology: -, negative; +, Unilateral Ground glass opacity (GGO)/Consolidation, ++, Bilateral GGO/Consolidation; +++, ARDS

## Results

**Fig. 1:**
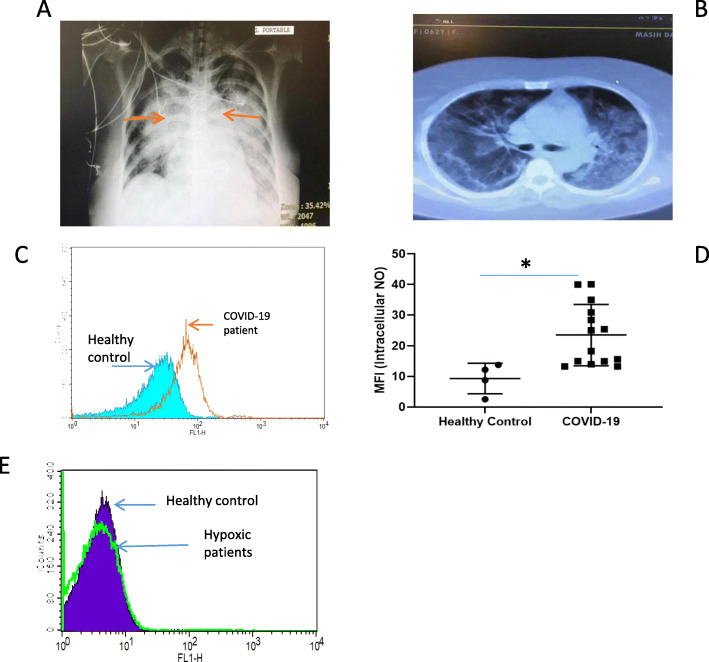
**a** Representative chest X ray of a COVID-19 patient on mechanical ventilation showing bilateral consolidations (red arrows). **b** Spiral CT scan of a representative COVID-19 patient indicating multiple bilateral patchy ground glass infiltration. **c** Red blood cells were preincubated with 5 mM of DAF for 20 min at 37 °C in PBS containing 1% BSA in the dark. Intracellular NO was determined by FACS analysis. A representative histograph from one out of 14 COVID-19 positive patients and 4 healthy controls is shown. **d** The mean fluorescent intensity (MFI) of all the subjects in each group is presented (*p < 0.05 using Student’s t-test). **e** Representative histogram of intracellular NO from RBCs of a single hypoxic non-COVID-19 patient

Chest X ray and CT imaging of the lungs shows significant changes in the lungs with bilateral alveolar diffusion confirming consolidation of the lungs in these patients (Fig. 1 a and b). In addition, patients with severe COVID-19 had higher serum lactate dehydrogenase (indicating tissue damage), C-reactive protein peaks (indicating inflammation) and lower counts of infection-fighting lymphocytes than those with milder disease. No significant differences were found in the levels of white blood cells, creatinine kinase (measuring muscle inflammation) or in lactic acid (measure of muscle oxygen levels) (Table 1). Having confirmed the clinical and radiographic features of COVID-19 in these subjects we then examined the levels of intracellular NO within their circulation RBCs.

Isolated RBC were stained with DAF and intracellular NO determined. COVID-19 patients had a rightward shift in the FACs plot reflecting higher intracellular levels of NO (Fig. 1c). The mean florescent intensity (MFI) was calculated for each subject and plotted as a histogram (Fig. 1d). This showed a significant increase in intracellular NO levels in RBCs from COVID-19 subjects (*P* ≤ 0.05).

To determine whether hypoxia itself may be responsible for the increased levels of intracellular NO in COVID-19 patient’s RBC, we examined intracellular NO levels of RBC from hypoxic patients without COVID-19 (due to COPD and emphysema). As depicted Fig. 1e, no significant increase of intracellular NO was seen in hypoxic patients compared to non-hypoxic controls.

## Discussion

We demonstrate increased levels of intracellular NO in RBC from COVID-19 subjects. This is not due to the presence of hypoxia per se but may afford protection against the hypoxia seen in COVID-19 patients. During health, constitutive NO production in RBCs is largely NOS-dependent, whereas in hypoxic conditions NO production may involve nitrite reduction by deoxyhemoglobin carbonic anydrase and/or eNOS itself [14].

RBC-derived NO causes the vasodilation of small vessels allowing oxygen to be readily released to tissues. In our study, intracellular RBC NO of COVID-19 patients is significantly higher than in healthy controls and this may enable the release of oxygen to tissues resulting in the clinical manifestation of silent hypoxia in these patients. Pronounced arterial hypoxemia without proportional signs of respiratory distress is reported in COVID-19 patients [15–18]. For example, Tobin and colleagues recently reported three cases of silent hypoxemia with a PaO2 ranging between 36 and 45 mmHg in the absence of increased alveolar ventilation [16].

However, the mechanism(s) underlying this silent hypoxia have not been explored despite the need to understand why some COVID-19 patients are able to continue with their normal daily activities despite often pronounced hypoxia [19].

Many theories have been proposed to account for this silent hypoxia. For example, silent hypoxia may be due to the differential effect of O_2_ and CO_2_ on gas exchange which may produce a relative preservation of the lungs’ ability to excrete CO_2_ despite falling O_2_ levels. Since the body is better able to detect changes in CO_2_ than O_2_, the relatively normal CO_2_ levels may attenuate any drive to increase the patients breathing rate despite the presence of low oxygen levels and thereby prevent the sensation of shortness of breath.

The mechanism(s) underlying NO generation inside RBC is not well understood. However, acidosis, hypoxemia and tissue hypoxia lead to NO generation by RBC via SNO–protein transfer of NO activity [20, 21]. The efficiency of NO produced by RBC NOS to promote vasodilation is not well described however perfusion of blood vessel segments with pre-sheared RBC suspensions caused a significant dilation under hypoxic conditions, but not high oxygen, levels [22]. Vasodilation was abolished by pre-incubation of the RBC suspension with the NOS inhibitor L-NAME. These findings support the concept that RBC-derived NO has a functional role in the regulation of local blood flow [22]. Moreover, shear stress induces ATP release from hypoxic RBC as a consequence of their role as O_2_ sensors [21].

Since NO is a pulmonary vasodilator and also has antiviral activity against coronavirus strains it is likely that exogenous NO treatment may be effective in COVID-19 subjects. There is no evidence that direct oxygen therapy is beneficial in the management of breathlessness in severe COVID-19 patients but our data suggests that NO therapy may be beneficial in COVID-19 patients with hypoxia [23].

Autoimmune hemolytic anemia (AIHA) was recently described in COVID-19 patients [24, 25]. AIHA causes platelet cell death and RBCs can also modulate platelet activity directly through either chemical signalling or direct RBC-platelet interactions. In this way RBCs promote platelet aggregation and degranulation by releasing ATP and ADP under low pO_2_, low pH and in response to mechanical deformation [26, 27]. In addition, the release of extracellular hemoglobin can also cause platelet activation by lowering NO bioavailability [28]. Thus, our current finding and evidence for hemolysis in patients may account for the microvascular coagulation seen in COVID-19 patients. We were unable to explore the mechanism(s) causing the accumulation of intracellular NO in RBC of COVID-19 patients in this study but this will be the focus of future research.

In summary, COVID-19 patients show higher levels of NO inside RBC compared to non-COVID-19 hypoxemic patients. Whether higher levels of intracellular NO inside RBC of COVID-19 infected patients drive the unexpected silent hypoxia phenotype needs to be examined in future clinical studies using NO donors in hypoxemic COVID-19 patients.

## Conclusions

This pilot study shows that elevated levels of intracellular NO may mask the effects of hypoxia in COVID-19 patients that presents as a silent hypoxic state. Further studies are required to confirm this but the data suggests that trials of NO therapy or NO donors may be useful in treating severe COVID-19 patients with hypoxia.

## Data Availability

The data will be available upon written request.

## Abbreviations

ARDS: Acute Respiratory Distress Syndrome (ARDS)
COVID-19: coronavirus disease 2019 (COVID-19
FACS: Fluorescence-activated cell sorting
MFI: Mean florescent of intensity
NO: Nitric oxide
RBC: Red blood cells
