# Correction to: Silent hypoxia: higher NO in red blood cells of COVID-19 patients

**DOI:** 10.1186/s12890-020-01326-0

**Published:** 2020-11-10

**Authors:** Esmaeil Mortaz, Majid Malkmohammad, Hamidreza Jamaati, Parisa Adimi Naghan, Seyed Mohamad Reza Hashemian, Payam Tabarsi, Maohammad Varahram, Hamidreza Zaheri, Efsun Gonca Uğur Chousein, Gert Folkerts, Ian M. Adcock

**Affiliations:** 1 Clinical Tuberculosis and Epidemiology Research Center, National Research Institute of Tuberculosis and Lung Diseases (NLRTLD), Shahid Beheshti University of Medical Sciences, Tehran, Iran; 2 Tracheal Disease Research Center, National Research Institute of Tuberculosisand Lung Diseases (NRITLD), Shahid Beheshti University of Medical Science, Tehran, Iran; 3 Chronic Respiratory Diseases Research Center, National Research Institute ofTuberculosis and Lung Diseases (NRITLD), Shahid Beheshti University of Medical Sciences, Tehran, Iran; 4 Mycobacteriology Research Center, National Research Institute of Tuberculosis and Lung Diseases (NRITLD), Masih Daneshvari Hospital, Shahid Beheshti University of Medical Sciences, Tehran, Iran; 5 University of Health Sciences Turkey, Yedikule Chest Diseases and Thoracic Surgery, Education and research Hospital, Department of pulmonology, Istanbul, Turkey; 6 Division of Pharmacology, Utrecht Institute for Pharmaceutical Sciences, Faculty of Science, Utrecht University, Utrecht, Netherlands; 7 Cell and Molecular Biology Group, Airways Disease Section, Faculty of Medicine, National Heart and Lung Institute, Imperial College London, London, UK; 8 Priority Research Centre for Asthma and Respiratory Disease, Hunter Medical Research Institute, University of Newcastle, Newcastle, NSW Australia

**Correction to: BMC Pulm Med 20, 269 (2020)**

**https://doi.org/10.1186/s12890-020-01310-8**

Following publication of the original article [1], the authors reported an error concerning the affiliation of the first and the sixth author, Esmaeil Mortaz and Payam Tabarsi.

They were affiliated with the following affiliation: Mycobacteriology Research Center, National Research Institute of Tuberculosis and Lung Diseases (NRITLD), Masih Daneshvari Hospital, Shahid Beheshti University of Medical Sciences, Tehran, Iran.

While they should be affiliated with this affiliation: Clinical Tuberculosis and Epidemiology Research Center, National Research Institute of Tuberculosis and Lung Diseases (NRITLD), Shahid Beheshti University of Medical Sciences, Tehran, Iran.

The original article [1] has been updated and the corrected affiliations are provided in this article (please see the reference list above).
